# Low-Dose Aspirin Recommendations for Preeclampsia Risk Reduction: A Systematic Review Focused on Black Patients: Current Hypertension Reports

**DOI:** 10.1007/s11906-026-01373-8

**Published:** 2026-03-20

**Authors:** Yitian Zha, Natalie J Mikhailov, Krishna H Brucia, Juan Gao, Emily Alleman, Keith C Ferdinand

**Affiliations:** 1 Tulane University School of Medicine, 1430 Tulane Avenue, New Orleans, LA 70112 USA; 2 Rudolph Matas Library of Health Sciences, Tulane University, New Orleans, USA

**Keywords:** Hypertension in pregnancy, Black race, Disparities, Low-dose aspirin, Preeclampsia prophylaxis, Paternal mortality

## Abstract

**Purpose:**

Daily low-dose aspirin (LDA) is guideline recommended for preeclampsia prevention, but racial/ethnic disparities persist in its implementation. This systematic review examines disparities in LDA prescription, recommendation and adherence for preeclampsia risk reduction among Black patients compared with other racial/ethnic groups in the United States.

**Recent findings:**

Overall, LDA prescription/recommendation rates range widely across included studies, from 10.7% to 95% among eligible patients. Black patients are shown to have a similar prescription/recommendation rate compared to White patients. Adherence rates average around 70%, with Black patients showing lower adherence rates despite similar prescription/recommendation rates than White patients. Barriers identified included inconsistent counseling, limited access to prenatal care, and distrust of the healthcare system.

**Summary:**

While prescribing practices for LDA appear increasingly equitable across racial/ethnic groups, significant disparities remain in adherence among Black women, potentially contributing to worse maternal outcomes. Reducing these gaps requires culturally tailored patient education, improved physician–patient communication, and systems-level interventions that integrate adherence monitoring into prenatal care.

## Introduction

Maternal mortality in the United States has increased from 17.4 per 100,000 live births in 2018, to 18.6 in 2023 despite advances in obstetric care [1, 2]. Black women bear a disproportionate burden of this crisis, experiencing maternal mortality rate over three times higher than their White counterparts [3]. Preeclampsia is a pregnancy-specific multiorgan system inflammatory syndrome characterized by new-onset of hypertension after 20 weeks of gestation accompanied by proteinuria or signs of other target organ dysfunction. In the setting of preeclampsia, hypertension is defined as systolic blood pressure (SBP) ≥ 140 mmHg or diastolic blood pressure (DBP) ≥ 90 mmHg on two separate occasions at least 4 h apart in a previously normotensive woman [4]. Overall, preeclampsia remains one of the leading causes of maternal death in the United States and represents a critical target for prevention intervention.

According to the 2025 American Heart Association/American College of Cardiology and Multisociety Guideline (2025 AHA/ACC/HBP Guideline) for the Prevention, Detection, Evaluation, and Management of High Blood Pressure in Adults, preeclampsia and overall hypertension in pregnancy should be a major concern for clinicians and public health [4]. The 2025 AHA/ACC/HBP Guideline supports the 2020 American College of Obstetricians and Gynecologists (ACOG) guideline for the definition of preeclampsia [5].

Moreover, eclampsia and hemolysis, elevated liver enzymes, and low platelet count (HELLP) syndrome are both severe complications of preeclampsia to which the disorder can progress. Both complications sometimes appear without preceding hypertension or proteinuria [6]. Eclampsia is the development of new-onset tonic-clonic, focal, or multifocal seizures, and can further lead to maternal complications such as hypoxia, trauma, aspiration pneumonia, and even death [7]. Beyond immediate risks, patients with preeclampsia face elevated long-term cardiovascular morbidity, including chronic hypertension, coronary heart disease, and stroke [8].

Given the substantial morbidity and mortality associated with preeclampsia, evidence-based prevention is essential. In 2014, the U.S. Preventive Services Task Force (USPSTF) recommended daily low-dose aspirin (LDA) prophylaxis after 12 weeks of gestation for pregnant individuals at high risk of preeclampsia and suggest its use in those with more than one moderate-risk factor [9]. Significantly, the 2021 USPSTF updated guideline recommended daily LDA prophylaxis for preeclampsia prevention in pregnant individuals, both at high risk and having more than one moderate-risk factor [10]. In this updated guideline, the USPSTF also refined one of its moderate-risk factor categories by separating the previous sociodemographic characteristics factor into two distinct factors: Black persons (due to social, rather than biological, factors) and lower income [10]. Moreover, ACOG and the Society for Maternal-Fetal Medicine (SMFM) similarly endorse daily LDA for preeclampsia prevention in at-risk populations [11].

Despite clear guideline recommendations and evidence supporting LDA prophylaxis for preeclampsia risk reduction, racial/ethnic disparities in preeclampsia incidence and maternal morbidity and mortality persist [3]. Black women experience higher rates of preeclampsia; worse maternal and perinatal outcomes compared with other racial/ethnic groups [12]. Whether these disparities reflect inequitable access to preventive therapies, differential adherence, or systemic healthcare barriers remains inadequately characterized. Although some studies included in this review distinguished non-Hispanic Black from Hispanic Black patients and non-Hispanic White from Hispanic White patients, most did not report ethnicity separately. Reporting of race and ethnicity in medical and health research can be inconsistent, potentially causing confusion and unintentional bias [13]. In consideration of these factors, this review uses the terms Black and White patients, without specifying ethnicity to maintain consistency with the included studies and adhere to being inclusive. Therefore, this systematic review examines racial/ethnic disparities as a general term in LDA prescription, recommendation, and adherence among Black women at risk for preeclampsia compared with other racial/ethnic populations in the United States.

## Methods

### Literature Search

This review was conducted to align with Preferred Reporting Items for Systematic Reviews and Meta-Analyses (PRISMA) guidelines. One author wrote the protocol and registered it with Prospero. A health sciences librarian (EA) developed the search strategy in PubMed and translated the search to two Elsevier databases: Embase and Scopus. The following keywords and corresponding subject headings (via MeSH and Emtree) were utilized in the search: preeclampsia, aspirin, and racial/ethnic disparities. A range of synonyms were used to describe each keyword: pregnancy-induced hypertension, gestational hypertension, pregnant elevated blood pressure, 2-(acetyloxy) benzoic acid, non-steroidal anti-inflammatory agents, over the counter pain medication, race factor, minority populations, health disparate populations, racial/ethnic /ethnic discrimination, or structural racism. A combination of the Boolean operators AND/OR was used. The librarian initiated the search on November 5, 2024 and concluded the search on January 24, 2025.

## Eligibility Criteria

This systematic review included studies investigating LDA use for preeclampsia prevention with a focus on racial/ethnic groups without a time limit. The search looked for peer-reviewed studies, written in English, and conducted in the United States. Quantitative observational studies – such as cohort, cross sectional, or case control studies – were included in the search. Systematic reviews and case reports were omitted. Inclusion criteria include (1) written in the English language, (2) conducted within the United States, (3) assessed LDA use as a preventive measure for preeclampsia, and (4) measured prescription or recommendation rate of LDA. Exclusion criteria include (1) not providing data relevant to LDA use for preeclampsia prevention, (2) studies that are case reports, commentaries, or reviews lacking primary data, and (3) articles that do not include racial/ethnic information about their data.

## Study Selections and Data Extraction

The screening process was conducted using Covidence systematic review software. Each title and abstract of all retrieved articles were reviewed by two of the four authors (YZ, KHB, NJM and JG) who conducted the study selection to determine if inclusion criteria were met. For each included abstract, a full-text article was obtained for review. Each full-text article was screened by two of the four authors (YZ, KHB, NJM and JG) independently. Full-text articles that met the inclusion criteria were included in this systematic study. Disagreements or discrepancies during the screening process were resolved by a third author. Data was extracted from each included full-text articles by two of the four authors (YZ, KHB, NJM and JG), and disagreements were resolved by a third author.

## Risk of Biases Assessment

The quality of included studies and risk of bias were assessed by using the Newcastle-Ottawa Scale [14].

## Results

### Study Selection

The initial search yielded 2,582 records across Embase (*n* = 1,923), Scopus (*n* = 518), and PubMed (*n* = 141). After the removal of 413 duplicates, 2,169 titles and abstracts were screened. Of these, 2,029 were excluded as irrelevant.

**Fig. 1:**
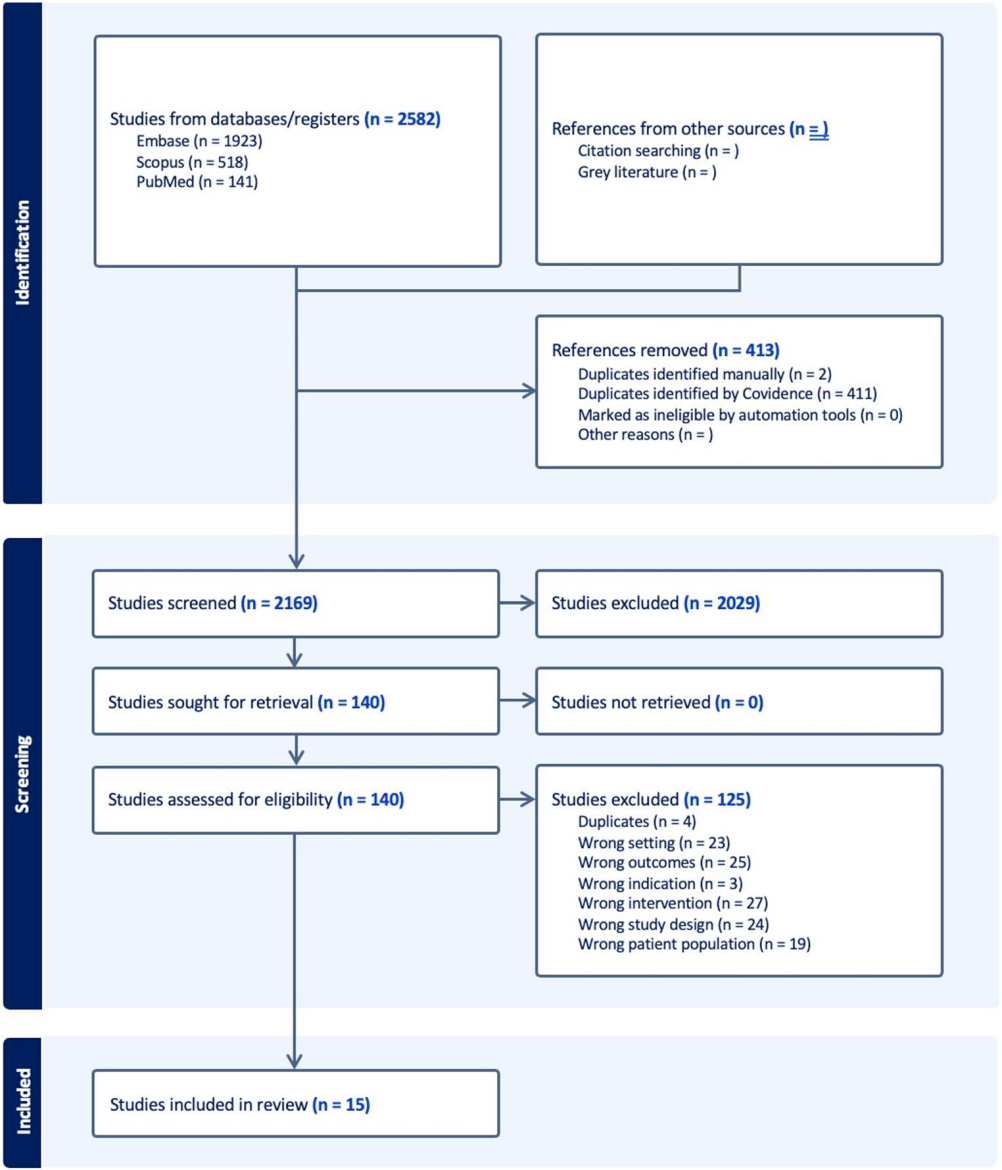
Study selection diagram. Flow diagram that includes searches of databases and registers

A total of 140 full-text articles were assessed for eligibility. Of these, 125 were excluded for the following reasons: wrong setting (*n* = 23), wrong outcomes (*n* = 25), wrong indication (*n* = 3), wrong intervention (*n* = 27), wrong study design (*n* = 24), wrong patient population (*n* = 19), and duplicates (*n* = 4). Ultimately, 15 studies met the inclusion criteria and were included in the final review. No additional studies were identified through citation searching or grey literature. A PRISMA flow diagram summarizing the selection process is provided in Fig. 1.

## Characteristics of Included Studies

The 15 included studies were conducted in the United States and published between 2020 and 2024. Study designs included retrospective cohort studies (*n* = 12), one quality improvement project, one non-randomized experimental study, and one cross-sectional study. Settings of research included urban safety-net hospitals (*n* = 3), tertiary care centers (*n* = 7), one federally qualified health center, and multi-hospital systems (*n* = 4). The populations studied were racially and ethnically diverse, with sample sizes ranging from 110 to 15,167 participants. Most of the included studies (*n* = 14) reported outcomes stratified by race, enabling direct comparison of LDA prescription/recommendation rates across racial/ethnic groups.

## Overall Prescription/Recommendation Rates

**Fig. 2:**
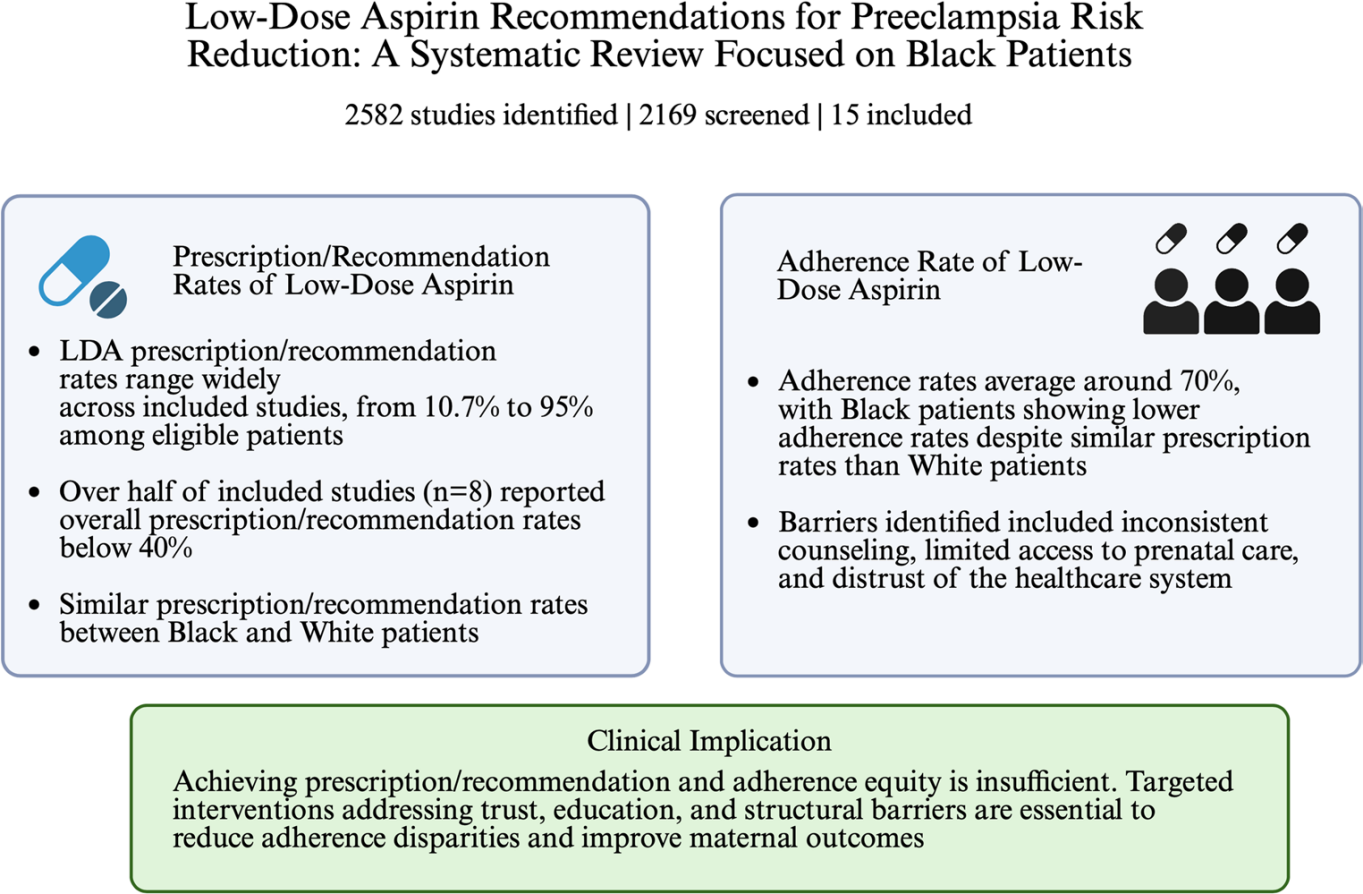
Central illustration summarizing the main findings of this systematic review

As demonstrated in Fig. 2, the overall LDA prescription/recommendation rates varied widely across included studies, ranging from 10.7% (Ayyash 2023, *n* = 15,167) to 95.0% (Boelig 2021 post-intervention, *n* = 136), with a mean rate of 39.2% [15, 16]. Over half of these studies (*n* = 8) reported overall prescription/recommendation rates below 40%, suggesting current under-prescribing even with guideline recommendations of LDA for preeclampsia prevention [15, 17–23]. The rest of the included studies (*n* = 7) reported overall prescription/recommendation rates over 50% [16, 24–29]. The two largest studies of included studies, which together comprised over half of the total study population (Ayyash 2023, *n* = 15,167; Freret 2024, *n* = 13,321), reported low LDA prescription/recommendation rates of 10.7% and 12.2% respectively, suggesting that missed opportunities for LDA prophylaxis remain common even in recent years [15, 19].

### Racial/ethnic Disparities in LDA Prescription/Recommendation and Uptake

Out of the 15 studies included in this review, there are five studies (Abbott 2020, Boelig 2021, Campo 2023, Lauring 2020, Mesina 2024) reported Hispanic/Latino as a race category, listed alongside White, Black, and Other [16, 17, 23, 28, 30]. Two studies (Singh 2023, Teal 2023) reported ethnicity in addition to race and used terms such as non-Hispanic Black and non-Hispanic White when describing the patient populations [21, 22]. However, the remaining eight studies (*n* = 8) didn’t specify the ethnicity of the Black and White populations included.

Among studies with reported prescription/recommendation rates stratified by race and ethnicity, findings regarding racial/ethnic disparities were mixed. For Black patients, LDA prescription/recommendation rates ranged from 10.4% to 80.0%, compared to 3.6% to 79% for White patients (Fig. 2). The majority of these studies showed similar prescription/recommendation rates between Black and White patients [26]. One study with no direct report on LDA prescription/recommendation rates based on race or ethnicity has commented that there was no statistically significant difference in LDA prescription/recommendation by race/ethnicity (*P* = 0.52) [30].

Two studies documented significantly lower prescription/recommendation rates for Black patients compared to White patients. Mesina 2024 (*n* = 714) reported that Black patients were 52% (*p* < 0.001) less likely than White patients to be prescribed LDA (OR 0.489, 95% CI [0.32–0.73]) [28]. Similarly, Wong 2022 (*n* = 677) found that only 10.4% of Black patients received LDA recommendations compared to 26.5% of non-Black patients [18].

### Adherence and Persistence

As shown in Fig. 2, among studies that reported adherence rate (*n* = 2), findings were consistent. Both studies reported lower adherence rates among Black women (65.4% and 63.8%) compared with White/other women (76.8% and 79%) [24, 29]. Barriers to adherence identified in several studies included inconsistent counseling, limited prenatal care access, and structural healthcare inequities. Where adherence was measured longitudinally, discontinuation rates were consistently higher among Black women, often attributed to unclear clinician guidance or limited understanding of aspirin’s preventive role [24, 29].

### Clinical Outcomes

A subset of studies evaluated whether disparities in LDA utilization were associated with differences in maternal and neonatal outcomes. Nevertheless, results were mixed, and some analyses indicated that lower LDA use among women who self-identified as Black or Hispanic which correlated with higher rates of preeclampsia and adverse perinatal outcomes, while others reported no significant differences once confounders were controlled. Importantly, multiple studies emphasized that reduced LDA initiation and poor adherence to LDA therapy in minority populations may exacerbate preexisting disparities in maternal morbidity.

Several studies included in this review evaluated the relationship between LDA use in pregnant women and clinical outcomes, including increased rates of preeclampsia and adverse perinatal events. Studies examining population-level implementation of LDA protocols have demonstrated that increased LDA use is associated with reductions in preeclampsia incidence and severity, including decreased rates of preterm preeclampsia and hypertensive complications of pregnancy.

Importantly, disparities in LDA initiation and adherence may contribute to persistent racial/ethnic differences in maternal outcomes. Some studies reported that lower LDA utilization among Black and Hispanic patients was associated with higher rates of preeclampsia and adverse neonatal outcomes, including preterm birth and low birth weight. However, these associations were not consistently observed across all studies, and several analyses found that racial/ethnic differences in outcomes were attenuated after adjusting clinical and socioeconomic confounders.

Overall, the available evidence suggests that LDA is an effective preventive intervention for preeclampsia. Nevertheless, inequities in its implementation, particularly in adherence, may contribute to ongoing disparities in preeclampsia-related morbidity. Further prospective studies are needed to directly link adherence patterns with clinical outcomes across diverse populations. 

### Synthesis of Evidence

Overall, the evidence highlights a persistent and concerning gap in overall implementation of guideline-based LDA prophylaxis. Women from certain racial/ethnic populations, particularly Black women, may face systemic barriers to maintaining consistent use of LDA, which may contribute to disparities in preeclampsia-related outcomes.

## Discussion

By examining disparities in LDA prescription/recommendation and utilization among populations at risk, this review aims to inform interventions improving maternal health outcomes and addressing the disproportionate burden of preeclampsia on Black women. Fifteen studies from the systematic review evaluated physician adherence to the 2014 USPSTF guideline or 2021 update recommending daily LDA for preeclampsia prevention in pregnant patients, as well as racial/ethnic disparities in prophylaxis recommendations [9, 10].

Therefore, this review has shown that Black women are prescribed LDA for preeclampsia prevention at rates similar to women from other racial/ethnic populations when risk factors are present, but Black patients’ rates of adherence are consistently lower. This finding is in line with a recent study by Vinogradov et al. that identified multiple obstacles to pregnant patients’ adherence, including insufficient knowledge about aspirin’s benefits, concerns about necessity and safety, access issues, social influences, as well as lack of habit formation [31]. These barriers are even more noticeable in marginalized populations, including Black women. It is important to mention that these factors are exacerbated by systemic issues such as distrust of the healthcare system and poor physician-patient communication. Similarly, a report from SMFM highlighted that nonadherence often stems from inconsistent counseling, mixed physician messaging, and logistical barriers, despite the low cost of LDA treatment [32].

It is also important to mention purposeful avoidance of LDA usage by some pregnant women. Some patients are cautious about taking LDA as prescribed or recommended due to potential risks of increased bleeding, and consequently, cerebrovascular accidents. However, a recent study by Cloud et al. notes that caution in aspirin use should only be exercised in older individuals who are prone to head trauma due to falls [33]. Young women of reproductive age are generally not included in this high-risk category.

The potential harm of any prescription drug is at the forefront of the minds of many expectant mothers, and even the most harmless and well-studied medications can raise concerns when the health of the future child is at stake. A recent public and scientific discussion about a potential link between the use of acetaminophen (Tylenol) during pregnancy and an increased risk of autism spectrum disorder (ASD) in children is one of the examples of such discourse. The overwhelming consensus among major medical and health organizations including ACOG, the Food and Drug Administration (FDA), and the World Health Organization (WHO), is that no established causal link between maternal acetaminophen usage during pregnancy and ASD in offspring [34, 35]. However, these statements are sometimes still not enough to convince a worried parent. A similar, though less widespread, hesitancy exists with respect to LDA usage in pregnancy and its link to ASD or attention deficit/hyperactivity disorder in offspring. Reassuringly, a recent study that came out in 2025 by Rodriguez-Sibaja et al. demonstrated no increased risk of maternal, fetal, or neonatal adverse events associated with LDA use during pregnancy [36]. This evidence reinforces current guideline recommendations and further confirms the need to address misconceptions that may contribute to intentional LDA nonadherence.

Moreover, we found that the reported prescription/recommendation rates of LDA in Black women vary across studies. Several studies suggest that the prescription/recommendation rates of LDA among Black women were lower than White women, even when the risk factors were present. In contrast, a few studies suggest equal or even higher prescription/recommendation rates of LDA among Black women, even though the adherence rates in these populations were statistically lower compared to White women.

Overall, the average LDA adherence rate among high-risk populations was about 70%. This important finding suggests that over one out of four pregnant women who are prescribed or recommended LDA do not use it consistently, despite strong guideline recommendations. It was also discovered that the LDA adherence rates among Black patients were consistently lower than among White patients, even though prescription/recommendation rates were similar. Prescription uptake itself was more comparable, with average rates in the 70–80% range across groups when risk factors were present. However, patient follow-through after prescription appears to be the most significant challenge.

To our knowledge, no previous studies have comprehensively examined LDA prescription/recommendation and adherence patterns in high-risk pregnancies by race/ethnicity, specifically comparing pre- and post-2014 USPSTF guideline adoption periods. This remains a critical gap in literature, and we aim to address this gap in our study. The literature highlights the underrepresentation of Black women in clinical trials, which limits the generalizability of data and emphasizes the need for research on implementation and adherence within this population. The study by Mendoza et al. determined that over 92% of participants in a recent large aspirin trial were White, explicitly stating that this lack of racial/ethnic diversity limits the applicability of results to Black women, who have a higher incidence of preeclampsia [37]. When analyzing the 2014 USPSTF guidelines for adoption, it appears that adherence had not improved significantly, highlighting a persistent gap in implementation. Some studies also reported that Black women were more likely to discontinue aspirin prematurely compared to White women, often citing inconsistent guidance from healthcare clinicians and the overall lack of understanding of the importance of LDA.

These findings highlight discrepancy between issuing the guidelines, prescription practices, and patient adherence. According to our analysis, physicians are generally following the guideline recommendation in prescribing aspirin to eligible pregnant patients of all racial/ethnic backgrounds, including Black women. Low adherence rates across high-risk patient groups, and particularly lower rates among Black women, suggest that current interventions are insufficient and must go beyond simple counseling and prescribing to increase patient adherence. Patient-centered interventions, such as trust-building approaches, culturally tailored education materials, and focused counseling should be prioritized by clinicians. Structural support tools such as reminder systems, pharmacy access programs, and integration of aspirin adherence into routine prenatal care monitoring may also be required. These findings align with maternal health disparities research showing that systemic inequities, such as structural racism, lower access to high-quality care, implicit bias, and adverse social determinants of health, and not just individual patient factors drive differences in outcomes [38]. The disproportionately high rates of preeclampsia among Black women cannot be reduced without addressing the combined effects of mistrust, communication barriers, and underrepresentation in clinical evidence-based research.

There were several limitations noted related to this study. First, the included studies were heterogeneous in design, methodology, and patient population which limited our ability to conduct a formal meta-analysis and required us to rely on descriptive interpretation of the data. Second, reporting of race and ethnicity was inconsistent across the studies, with some authors combining groups or omitting data on patients of other racial/ethnic backgrounds. Third, this review relies on retrospective data and data from electronic health records in many studies, which may underestimate aspirin counseling or adherence due to incomplete documentation. Similarly, adherence measures were often based on data about prescription fills or self-report, both of which may not accurately reflect actual medication use and fall short due to recall bias. Fourth, because the studies included the span of pre- and post-2014 and 2021 USPSTF guideline adoption, temporal differences in physician practice and patient uptake could confound comparisons. Finally, women of Asian population were not included in this study. Due to data limitations, majority of the studies did not report data on Asian patients. Within the few studies that did, the results of LDA prescription/recommendation rates were similar in Asian patients as seen in White patients.

There are a few critical avenues for improvement in understanding LDA prescribing and adherence in racially and ethnically diverse pregnant populations. These avenues include conducting large-scale prospective studies that separate outcomes by race and ethnicity, developing standardized methods to measure adherence that go beyond prescription fill or self-report data, and identifying which factors most strongly predict continuous use of aspirin during pregnancy. A recent review has demonstrated that universal risk assessment tools, electronic health record prompts, and structured clinician education can improve prescribing practices, but future randomized implementation studies are necessary in larger, more diverse samples to better demonstrate longitudinal impacts on patient adherence and maternal health outcomes [39].

Furthermore, community-based studies that examined adherence behaviors and maternal outcomes in diverse settings have suggested that culturally tailored education and patient-centered counseling can improve medication uptake [40]. However, more research is needed to determine which social, demographic, or clinical factors most strongly predict sustained adherence to aspirin use across different racial/ethnic groups. Alternative approaches, such as including adherence monitoring into routine prenatal visits and using mobile health phone applications for reminders, have also demonstrated promise in improving consistent use of medications during pregnancy [41]. These community-based strategies can be further combined with health education modules to address mistrust, barriers to care, and current knowledge gaps.

Another approach to reducing racial/ethnic disparities in preeclampsia outcomes is to focus on comprehensive systems-level interventions. Integrated prenatal care models and multidisciplinary maternal health programs have demonstrated improvements in hypertension management and maternal outcomes, by expanding access, improving continuity of care, and strengthening physician–patient communication [42]. Adding LDA adherence monitoring within such models may further reduce racial/ethnic disparities. In addition, future research should evaluate biological and pharmacogenomic factors that may influence aspirin effectiveness across populations, given the higher baseline risk of preeclampsia among Black women.

Establishing standardized reporting frameworks for LDA prescribing and adherence rates, stratified by race and ethnicity, will also be essential to track progress and guide evidence-based policy updates. Since the completion of this review, several new studies on LDA use for preeclampsia prevention have been published in 2025. A recent paper published in October 2025 by Jaclyn Del Pozzo discussed the utilization of a universal LDA protocol for preeclampsia prevention with retrospective cohort studies pre- and post-USPSTF guidelines update in 2021 [43]. They found this implementation of universal LDA protocol in a high-risk, underserved population markedly improved LDA adherence and reduced severe preeclampsia without increasing hemorrhage risk. Another retrospective cohort study analyzing the LDA use before and after the 2021 USPSTF guideline update came out in May 2025, and they found overall rates of LDA use remain well below expected even with the updated guidelines emphasizing racial/ethnic inequities of preeclampsia [44].

## Conclusion

This review concisely summarizes current evidence on LDA prescribing patterns for preeclampsia prophylaxis among Black patients. Existing literature demonstrates that patients who identify as Black receive LDA prescription/recommendation at a lower or similar rate than those who identify as White. However, adherence to LDA is consistently lower among Black patients than White patients, highlighting persistent racial/ethnic disparities that demand targeted interventions. It is important to emphasize that additional to updating guidelines, improving the quality and equity of maternal healthcare in the United States requires rigorous and consistent implementation of these evidence-based recommendations.

This systematic review provides a valuable knowledge base that raises awareness among clinicians and highlights the urgent need to improve patient adherence in practice. Its findings can inform interventions in several key areas, such as enhancing patient education within Black communities, improving drug-taking notification systems, and strengthening trust relationships between physicians and patients.

## Key References

- Writing Committee Members, Jones DW, Ferdinand KC, Taler SJ, Johnson HM, Shimbo D, et al. 2025 AHA/ACC/AANP/AAPA/ABC/ACCP/ACPM/AGS/AMA/ASPC/NMA/PCNA/SGIM Guideline for the Prevention, Detection, Evaluation and Management of High Blood Pressure in Adults: A Report of the American College of Cardiology/American Heart Association Joint Committee on Clinical Practice Guidelines. Circulation. 2025 Sept 16;152(11):e114–218. 
  - ○  This is the most current guideline for high blood pressure management with specific guidance on hypertensive disorders of pregnancy. Aligns with ACOG definitions of preeclampsia and provides evidence-based framework for blood pressure thresholds and management strategies during pregnancy.
- Hoyert DL. Maternal Mortality Rates in the United States, 2023 [Internet]. 2025 [cited 2025 Oct 28]. Available from: https://stacks.cdc.gov
  - ○  These are the most recent national maternal mortality data demonstrating persistent racial/ethnic disparities. This report provides critical epidemiological context for the urgency of addressing preeclampsia prevention gaps, particularly among non-Hispanic Black women, who experience mortality rates nearly three times higher than White women.
- Rodriguez-Sibaja MJ, Galvez-Rubalcava N, Hagerman-Sucar G, Alcocer-Gonzalez Camarena P, Gomez-Woodworth JR, Villalpando-Juarez MI, et al. Maternal, fetal, and neonatal serious adverse events associated with low-dose aspirin during the first trimester of pregnancy: A secondary analysis of the Aspirin Supplementation for Pregnancy Indicated Risk Reduction In Nulliparas (ASPIRIN) trial. Am J Obstet Gynecol MFM. 2025 Nov;7(11):101768.
  - ○  This is a comprehensive meta-analysis confirming no increased risk of maternal, fetal, or neonatal adverse events with LDA use during pregnancy. This study provides evidence addressing patient safety concerns that may contribute to aspirin use hesitancy and nonadherence, providing reassurance for both clinicians and patients.
- Vinogradov R, Holden E, Patel M, Grigg R, Errington L, Araújo-Soares V, et al. Barriers and facilitators of adherence to low-dose aspirin during pregnancy: A co-produced systematic review and COM-B framework synthesis of qualitative evidence. PLOS ONE. 2024 May 3;19(5):e0302720.
  - ○  This systematic review recognizes barriers to aspirin adherence, including knowledge gaps, safety concerns, access issues, and healthcare system distrust. This work provides critical insight into the mechanisms underlying the adherence of disparities documented in this review.
- Ayyash M, Goyert G, Pitts D, Khangura R, Garcia R, Jacobsen G, et al. Provider adherence to aspirin prophylaxis prescription guidelines for preeclampsia. Pregnancy Hypertens. 2023 Dec 1;34:1–4.
  - ○  This cross-sectional analysis (n=13,321) shows a modest increase in LDA use among Black patients following 2021 guideline changes emphasizing racial/ethnic disparities (8.0% pre-guideline to 18.3% post-guideline). It documents the real-world impact of guidelines and persistent implementation gaps.

## Data Availability

No datasets were generated or analysed during the current study.
